# MCBT: Multi-Hop Cluster Based Stable Backbone Trees for Data Collection and Dissemination in WSNs

**DOI:** 10.3390/s90806028

**Published:** 2009-07-29

**Authors:** Inyoung Shin, Moonseong Kim, Matt W. Mutka, Hyunseung Choo, Tae-Jin Lee

**Affiliations:** 1 Convergence Lab. Digital Media & Communications R&D Center, Samsung Electronics, Korea; E-Mail: inyoung.shin@gmail.com; 2 Department of Computer Science and Engineering, Michigan State University, USA; E-Mails: mkim@msu.edu; mutka@cse.msu.edu; 3 School of Information and Communication Engineering, Sungkyunkwan University, Korea; E-Mails: choo@ece.skku.ac.kr; tjlee@ece.skku.ac.kr

**Keywords:** multi-hop cluster, backbone, load balancing, energy efficient routing, network lifetime

## Abstract

We propose a stable backbone tree construction algorithm using multi-hop clusters for wireless sensor networks (WSNs). The hierarchical cluster structure has advantages in data fusion and aggregation. Energy consumption can be decreased by managing nodes with cluster heads. Backbone nodes, which are responsible for performing and managing multi-hop communication, can reduce the communication overhead such as control traffic and minimize the number of active nodes. Previous backbone construction algorithms, such as Hierarchical Cluster-based Data Dissemination (HCDD) and Multicluster, Mobile, Multimedia radio network (MMM), consume energy quickly. They are designed without regard to appropriate factors such as residual energy and degree (the number of connections or edges to other nodes) of a node for WSNs. Thus, the network is quickly disconnected or has to reconstruct a backbone. We propose a distributed algorithm to create a stable backbone by selecting the nodes with higher energy or degree as the cluster heads. This increases the overall network lifetime. Moreover, the proposed method balances energy consumption by distributing the traffic load among nodes around the cluster head. In the simulation, the proposed scheme outperforms previous clustering schemes in terms of the average and the standard deviation of residual energy or degree of backbone nodes, the average residual energy of backbone nodes after disseminating the sensed data, and the network lifetime.

## 1. Introduction

Wireless sensor networks (WSNs) have a wide range of potential applications, including environment monitoring, military surveillance, and remote medical systems [1]. The energy-efficient routing has become a key issue attracting recent interest, since the battery of nodes is the most critical limiting factor [2–5]. Power failure of a node affects its ability to forward the data packets of others as well as sending its own messages. Thus, the overall network lifetime will be seriously reduced. For this reason, extensive research has focused on how to prolong the network lifetime [6–12].

Much of the research on energy efficient routing for WSNs has several drawbacks. A single-hop clustering algorithm [13] can reduce energy consumption since only a fraction of the nodes, called cluster heads, communicate with the sink in one hop. However, a single-hop clustering algorithm is impractical, because nodes at a distance greater than one hop from the sink do not have adequate transmission power to communicate with the sink [14]. Although the multi-hop model would be a more practical approach to solve this problem, it can increase communication overhead and costs required to obtain the routing information in large-scale networks. In view of these issues, routing through a backbone reduces communication overhead and overall energy consumption for WSNs [15–17]. However, backbone nodes require extra functionality and therefore consume more energy compared with other nodes in the network. Unbalanced energy consumption among the sensor nodes may cause network partition and node failures where transmission from some sensors to the sink node becomes blocked. Therefore, construction of a stable backbone is one of the challenges in sensor network applications [2].

*Definition 1* (*d*-hop cluster) - The *d*-hop cluster is the set of nodes that are at most *d* hops away from a cluster head. Each cluster has one cluster head and several cluster gateways and cluster members.

Hierarchical Cluster-based Data Dissemination (HCDD) [18] applies a backbone construction algorithm for wireless ad hoc networks, such as Max-Min *d*-cluster [19], to WSNs. The Max-Min *d*-cluster diffuses node IDs to neighbors and each node selects the cluster head within a maximum *d*-hop to transmit data efficiently. Note that backbone construction for ad hoc networks is not considered energy consumption. Therefore, this scheme is inadequate for WSNs that are sensitive to energy. The residual energy in WSNs changes based on data transmission. Moreover, the degree of the node could decrease due to energy exhaustion. In the frequently changing network topology, we need to use a routing method that reflects the current network conditions, such as residual energy and degree, rather than using fixed node IDs.

The Max-Min *d*-cluster forms clusters based on a pre-allocated ID, so there is a high probability of selecting the same node again as a cluster head when attempting to form new clusters. This characteristic decreases the transition overheads when old cluster heads give routing information to new cluster heads. However, the disadvantages are that the energy consumption of specific nodes increases since the same nodes are repeatedly selected. Therefore, in this paper, we present a novel algorithm to form a multi-hop cluster based stable backbone trees, called MCBT. This algorithm is appropriate for dynamic sensor networks by reflecting the network conditions such as residual energy and degree. It selects nodes with higher energy or degree as the cluster heads in order to prolong the network lifetime. Moreover, MCBT balances the energy consumption by distributing the packet transmission role among the nodes around the cluster head.

This paper is organized as follows: in Section 2, the major routing techniques are introduced and discussed. Section 3 presents our stable backbone formation scheme for WSNs. The performance of MCBT is evaluated in Section 4. Finally, we conclude with the main findings and contributions of our research in the last section.

## 2. Related Work

### 2.1. Traditional Energy Efficient Routing Protocols

In the single-hop communication model, each sensor node communicates with the cluster head within a single-hop, and the cluster head transfers the sensed data directly to the sink (base station). Low Energy Adaptive Clustering Hierarchy (LEACH) [6] is a cluster based protocol that randomly rotates cluster heads to evenly distribute the energy load among the sensor nodes in the network. LEACH is able to incorporate data fusion into the routing protocol to reduce the amount of information that must be transmitted to the sink. This largely reduces the energy dissipated since the computation requires a negligible amount of energy compared with communication. LEACH uses single-hop routing where the cluster head transmits directly to the sink. However, this is infeasible in large scale sensor networks comprised of thousands of sensor nodes, due to the limited transmission power of sensor nodes.

The multi-hop communication model [20, 21] is a more practical approach to solve this problem. In this model, data hops from one node to another until it reaches the sink. In view of the limited transmission range of the sensor nodes, this is a viable approach and most nodes can connect and transmit their packets to the sink. Therefore, the coverage area of the sensor nodes and the sink in this model is an improvement over the single-hop model. Sensor Protocols for Information via Negotiation (SPIN) [20] efficiently disseminates information in a multi-hop manner. Nodes running SPIN assign a high-level name to their data, called meta-data, and perform meta-data negotiations before any data is transmitted. This assures that there is no redundant data sent throughout the network. In addition, SPIN has access to the current energy level of the node and adapts the protocol for WSNs. However, since all nodes participate in the route discovery phase, the multi-hop routing protocols may cause data communication problems, such as broadcasting storm and unnecessary communication overhead. Consequently, applying these approaches to large-scale sensor networks might considerably increase the overhead and associated costs.

### 2.2. Routing Protocols through a Backbone

Routing through a backbone is restricted in a set of particular nodes to reduce the communication overhead for route discovery and the number of active nodes for WSNs. A typical approach for backbone formation is to partition the network into clusters consisted of cluster heads and ordinary nodes. The cluster heads are then linked to form the connected backbone. Several approaches have been presented to select the cluster heads and construct the clusters. The optimal selection of cluster heads is an NP-hard problem [22], thus many heuristic methods have been designed for approximate solutions. The clustering phase of heuristics can be done in both centralized and distributed ways. In our work, we are interested in the network clustering of large-scale distributed systems. The challenge is to partition the network in an efficient and distributed manner, i.e., to design an effective distributed clustering protocol.

Such protocols include (i) highest connectivity cluster algorithm and (ii) highest-ID or lowest-ID cluster algorithm. The highest connectivity cluster algorithm of MMM [23] creates the clusters to consider the node’s connectivity. Nodes with the highest connectivity are elected as cluster heads. In the case of a tie, the node with the lowest ID prevails. Other nodes will be members of the nearest cluster head. However, this approach can result in a high turnover of cluster heads as the network topology changes, which is undesirable due to the high overhead associated with cluster heads changeover. The lowest-ID cluster algorithm of MMM creates the clusters using the unique IDs of the nodes. The lowest-ID node among neighbors is elected as the cluster head. Other nodes could be organized using the same process of the highest connectivity cluster algorithm of MMM.

HCDD applies the backbone construction algorithm for wireless ad hoc networks, such as Max-Min *d*-cluster, to WSNs. It forms *d*-hop clusters using the node ID. The *d*-hop cluster method is efficient for large-scale networks because it generates an appropriate number of cluster heads and the sensed data is transmitted in a multi-hop manner. The Max-Min *d*-cluster balances the load of the cluster heads by allocating a similar number of nodes in each cluster. However, there are many characteristics in WSNs, such as limited node resources, battery dependency and data fusion. Thus, this method cannot be applied to a realistic sensor environment. The Max-Min *d*-cluster algorithm forms clusters using a randomly allocating node ID. It has a tendency to re-elect existing cluster heads when it reconstructs the backbone. In spite of having a heavier load than that of the ordinary node, the cluster head will consume energy quickly if it has low energy. As a result, the network is easily disconnected or must form a new backbone. The Max-Min *d*-cluster algorithm may be suitable for ad hoc networks in which nodes have high energy and higher mobility. However, when we apply it to WSNs, it needs to form a backbone considering appropriate factors, rather than considering the fixed node ID.

## 3. Multi-hop Cluster Based Stable Backbone Trees (MCBT)

### 3.1. Motivation and New Factor

Backbone nodes consume more energy than ordinary nodes. It is important to form a stable backbone by taking into account the node’s residual energy and degree in order to prolong the network lifetime. MCBT constructs a stable backbone by selecting nodes with higher energy or degree as the cluster heads and distributes the role of packet forwarding among nodes around the cluster heads to enhance the network lifetime. In this paper, we consider a new factor: the flooding value. Using the flooding value, we can simultaneously consider the residual energy and degree, which are the core factors to evaluate the stability of the sensor node. If the node has high residual energy and degree, it has a high flooding value.

*Definition 2* (Round) - Round is the stage in which each node exchanges flooding values with 1-hop neighbors, and chooses the flooding value among its own and the neighbors’ flooding values. Round 0 is the computation step of the flooding value and MCBT performs the (2*d* + 1) rounds to form the *d*-hop clusters.

*Definition 3* (FloodingArray) - FloodingArray is the (2*d*+1) sized array to record the selected flooding value for each round. The first element of FloodingArray is the flooding value in round 0. Flooding values selected from round 1 to round 2*d* are sequentially recorded in the array.

Each node exchanges the flooding values with its neighbors for 2*d* rounds to form *d*-hop clusters and selects the node with higher flooding value as the cluster head. In each round, all nodes broadcast the flooding values selected in the previous round to their 1-hop neighbors and select one value by comparing among its own flooding value and the flooding values received from neighbors. This process is repeatedly performed for 2*d* rounds. The algorithm selects the nodes with a higher flooding value as cluster heads, applying the cluster head selection rule based on all values saved in FloodingArray. Using the following criterion, the flooding value takes both the node’s residual energy and degree into account:

(1) $$f(i,\omega )=\omega \left(\frac{E_{\mathrm{res}}}{E_{\mathrm{ini}}}\right)+(1-\omega )\left(\frac{\deg_{i}}{{\text{max}}_{j\in S_{i}}\left(\deg_{j}\right)}\right)$$

where *S_i_* is a set of neighbors of node *i* including itself.

Let *f*(*i, ω*) denote the flooding value of the node *i*; *ω* is the weight factor that adjusts the priority and *ω ∈* [0, 1]. A large *ω* gives more weight to the node’s residual energy than its degree. *E_ini_* is the initial energy of a node and *E_res_* is the residual energy of the node *i*. The *deg_i_* is the number of nodes that are within the node *i*’s transmission range, namely its neighbors. The max*_j∈S_i__*(*deg_j_*) is the maximum degree among its own degree and the degrees of the neighbors of the node *i*. Note that $\frac{E_{\mathrm{res}}}{E_{\mathrm{ini}}}\in [0,\;1]$ and $\frac{\deg_{i}}{{\text{max}}_{j\in S_{i}}\;\left(\deg_{j}\right)}\in (0,\;1]$. Suppose $\frac{E_{\mathrm{res}}}{E_{\mathrm{ini}}}$ remains constant; in this case, the flooding value increases when the degree increases. On the contrary, suppose $\frac{\deg_{i}}{{\text{max}}_{j\in S_{i}}\left(\deg_{j}\right)}$ and *E_ini_* are constant; then, the flooding value increases as *E_res_* increases.

### 3.2. The Clustering Algorithm

The proposed cluster formation consists of three steps. First, each node calculates its own flooding value in round 0, as mentioned in the previous subsection, and maintains the (2*d* + 1) sized FloodingArray. Second, this is followed by the Floodmax and Floodmin stage; each stage is performed for *d* rounds in which each node propagates the flooding value to its *d*-hop neighbors. The elements from 1*^st^* to *d^th^* in the FloodingArray are the selected the flooding values in Floodmax and the rest from (*d* + 1)*^th^* to (2*d*)*^th^* in FloodingArray are the selected flooding values in Floodmin. Finally, after Floodmax and Floodmin, each node looks at the all elements in FloodingArray to best determine its cluster head. The detailed procedures of Floodmax and Floodmin in the second step are as follows:

*Definition 4* (Floodmax-*i*) - In round *i* (1 *≤ i ≤ d*) of Floodmax-*i*, each node broadcasts its flooding value in the round (*i* − 1) and selects the maximum value among its own flooding value and the flooding values received from neighbors, and records it as the *i^th^* element in FloodingArray. This process runs for *d* rounds.

*Definition 5* (Floodmax-*j*) - After Floodmax, Floodmin is performed for *d* rounds. In round *j* (*d*+1 ≤ *j* ≤ 2*d*) of Floodmin-*j*, each node broadcasts its flooding value in the round (*j* − 1) and selects the minimum value among its own flooding value and the flooding values received from neighbors, and records it as the *j^th^* element in FloodingArray.

In Floodmax, the nodes with the higher flooding value propagate their flooding value in the 0*^th^* round to their node’s *d*-hop neighbors. Therefore, the flooding values selected by each node in Floodmax-*d* are the maximum values in the overall network. The nodes with this flooding value are finally selected as cluster heads. However, if nodes with the same flooding value in Floodmax-*d* are grouped, there would be a heavy load on the node with the highest flooding value, because the number of cluster members of nodes with higher flooding value increases. Floodmin balances the cluster heads’ load by evenly distributing the number of cluster members. Floodmin has the similar process as Floodmax, but each node selects the minimum value among its own flooding value and the flooding values received from neighbors. At the conclusion of Floodmin, the flooding values selected in Floodmax-*d* are evenly distributed through the overall network by further propagating lower flooding values.

**Figure 1.**
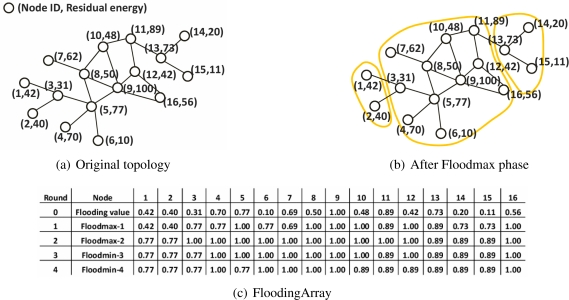
2-hop clustering - Floodmax and Floodmin phase.

**Figure 2.**
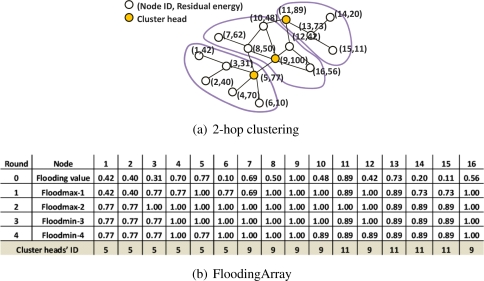
2-hop clustering - Resulting network topology.

Figure 1 shows a demonstration of Floodmax and Floodmin. Figure 2(a) presents the network topology with 16 nodes. For simplicity, it is assumed that the weight factor (*ω*) is 1 and the initial energy of each node is 100. Each node records the 0*^th^* element in FloodingArray by computing the flooding value using its residual energy and degree. In the case of node 3, the residual energy is 31, thus the flooding value is 0.31 according to Equation (1). In Floodmax-1, each node chooses the largest value among its own flooding value and the flooding values of 1-hop neighbors and records the selected flooding value as 1*^st^* element in its FloodingArray. Node 3 selects the highest value, 0.77 among 0.31, 0.42, 0.40, and 0.77, the flooding values in the 0*^th^* round of node 3 and its neighbors 1, 2, 5, respectively. In Floodmax-2, 1.00 is selected as the maximum value among its own flooding value and the flooding values of neighbors in Floodmax-1. Figure 2(b) shows the groups of nodes that select the same flooding value in Floodmax-2. Among the 16 nodes, 11 nodes select the highest flooding value in Floodmax-2, 1.00. Floodmax is a greedy algorithm and may result in an unbalanced load for the cluster heads. However, using Flood-min, we can evenly distribute the flooding values selected in Floodmax-*d* to the overall network. In Floodmin-3, each node selects the minimum value among the flooding value of the neighbors including itself in Floodmax-2. Node 3 selects 0.77 in Floodmin-3 and the same number in Floodmin-4. Nodes 3, 5, and 11 regain the flooding value of 0*^th^* round or the flooding value in Floodmax-1 by Floodmin.

After Floodmax and Floodmin, the node declares itself a cluster head or selects another node as a cluster head using three rules of cluster head selection.

***Rule 1***: If a node *x* has the same value in Floodmin-2*d* of the 0*^th^* round value, node *x* declares itself a cluster head and skip the rest of the rules.
***Rule 2***: If the first rule cannot be applied, node *x* compares *d* values of Floodmax with *d* values of Floodmin in its FloodingArray without order. If there is the value that appears in both Floodmax and Floodmin, node *y* that has this value is selected as the cluster head of node *x*. If node *x* has two or more of the same flooding values in its FloodingArray, it selects the node *y* that has a lower flooding value.
***Rule 3***: If node *y*’ flooding value in 0*^th^* round is the same to the value of Floodmax-d of node *x*, node *y* becomes the cluster head of node *x*.

After cluster head selection, each node broadcasts its elected cluster head’s ID to all of its neighbors. If a node receives a different cluster head ID, this node becomes a cluster gateway. Finally, in the case of a cluster head that is on the path between an ordinary node and its elected cluster head, the ordinary node chooses the cluster head with the minimum hops.

Applying the first rule, if the flooding value selected in Floodmax-*d* is same to the 0*^th^* round value, the node declares itself a cluster head. Two cases can be classified. First, in the case of node 9, its flooding value of the 0*^th^* round is the maximum value among the flooding values of the *d*-hop neighbors, so it continuously selects its flooding value. Second, with nodes 5 and 11, the flooding values in the 0*^th^* round are deleted from their FloodingArray, because they select higher flooding values in Floodmax. However, their *d*-hop neighbors transmit the flooding values of nodes 5 and 11 and save them in the FloodingArray because these flooding values are higher than the flooding values of their *d*-hop neighbors. Then the Floodmin process will successfully propagate these flooding values back to the originating nodes. To select the node that can satisfy the two cases, if the flooding value selected in Floodmin-2*d* is its own flooding value in the 0*^th^* round, this node will declare itself to be the cluster head.

Other nodes select their cluster head by the second and the third rule. Applying the second rule, the node selects one node among cluster heads within *d*-hop as its cluster head. Figure 2 shows that node 3 receives again the flooding value 0.77 by Floodmin. Therefore, 0.77 is the pair selected in both the Floodmax and Floodmin and node 3 selects node 5 with this value as the 0*^th^* round value as its cluster head. That is, node 3 selects the adjacent node 5, as a cluster head instead of node 9 that has a higher flooding value in the 0*^th^* round. As the result, the cluster head that has a lower flooding value can gain more cluster members. All nodes except nodes 5, 9 and 11 conform to the second rule of cluster head selection. The node to which the first and the second rule are not applicable selects the node with the flooding value in Floodmax-*d* as the 0*^th^* round value; that is, the maximum flooding value among *d*-hop neighbors. After the cluster head selection process, each node broadcasts its elected cluster head’s ID to all of its neighbors. Nodes 4 and 6 conform to the case of a cluster head being on the path between an ordinary node and its elected cluster head. Nodes 4 and 6 choose the cluster head with the minimum hops. Here it can be seen that there are three cluster heads (nodes 5, 9, and 11) whose energy is higher than that of the other nodes. Moreover, MCBT constructs three similar-sized clusters.

**Algorithm:** MCBT Clustering Algorithm **Input:** Undirected Graph(*G*), residual energy(*E_res_*), and *d* **Output:** Cluster heads’ ID

// FloodingArray*_i_*: FloodingArray of node *i*, CH*_i_*: Cluster head’ ID of node *i*	
**MCBT**(*G, E_res_, d*)	
**01:**	*n* ← The number of nodes
**02:**	**for***i* ← 1 **to***n*
**03:**	**do**$f(i,\omega )\leftarrow \omega \left(\frac{E_{\mathrm{res}}}{E_{\mathrm{ini}}}\right)+(1-\omega )\;\left(\frac{\deg_{i}}{{\text{max}}_{j\in S_{i}}\left(\deg_{j}\right)}\right)$,
	where *S_i_* is a set of neighbors of node *i* including itself
**04:**	FloodingArray*_i_*[0] ← *f*(*i, ω*)
// Floodmax Phase	
**05:**	**for***k* ← 1 to *d*
**06:**	**do for***i* ← 1 **to***n*
**07:**	**do** BROADCAST(FloodingArray*_i_*[*k* − 1])
	// A node broadcasts its (*k* − 1)*^th^* flooding value to neighbors and receive
	// all neighbors’ (*k* − 1)*^th^* flooding value
**08:**	**do for***i* ← 1 **to***n*
**09:**	**do** FloodingArray*_i_*[*k*] ← max*_j∈S_i__* (FloodingArray*_j_*[*k* − 1])
// Floodmin Phase	
**10:**	**for***k* ← 1 **to***d*
**11:**	**do for***i* ← 1 **to***n*
**12:**	**do** BROADCAST(FloodingArray*_i_*[*k* − 1 + *d*])
**13:**	**do for***i* ← 1 **to***n*
**14:**	**do** FloodingArray*_i_*[*k* + *d*] ← min_*j∈S*_*i*__ (FloodingArray*_j_*[*k* − 1 + *d*])
// Cluster head Selection	
**15:**	**do for***i* ← 1 **to***n*
**16:**	**do if** (FloodingArray*_i_*[2*d*]=FloodingArray*_i_*[0])
**17:**	**then** CH*_i_* ← *i*
**18:**	**else if**(Pair(*i*))
**19:**	**then** CH*_i_* ← CH_ID(Pair(*i*))
**20:**	**else** CH*_i_* ← CH_ID(FloodingArray*_i_*[*d*])
**Pair**(*id*)	
**01:**	**for***i* ← 1 **to***d*
**02:**	**do for***j* ← (*d* + 1) **to** 2*d*
**03:**	**do if** (FloodingArray*_id_*[*i*] = FloodingArray*_id_*[*j*])
**04:**	**then return** FloodingArray*_id_*[*i*]
**05:**	**return** 0
**CH_ID**(*floodingvalue*)	
**01:**	**for***i* ← 1 **to***n*
**02:**	**do if** (FloodingArray*_i_*[0] = *floodingvalue*)
**03:**	**then return***i*
**04:**	**return***error*

The pseudo code of MCBT clustering algorithm is shown above. Lines 1–4 show that each node computes its flooding value using the residual energy and degree, and makes a (2*d* + 1) sized FloodingArray. Lines 5–9 explain the process of Floodmax-*i* (1 ≤ *i* ≤ *d*). In round *i*, each node broadcasts its flooding value in the round (*i* − 1) and receives the neighbors’ flooding values. The node then selects the maximum value among its own flooding value and the flooding values received from neighbors. Each node records the selected flooding value as the *i^th^* element in its FloodingArray. Lines 10–14 explain the process of Floodmin-*j* (*d* + 1 ≤ *j* ≤ 2*d*), which is the same as Floodmax except a node chooses the smallest value rather than the largest value. In lines 15–20, each node declares itself a cluster head or selects another node as its cluster head based on the cluster head selection rules. Lines 16–17 describe the first cluster head selection rule. If the element in FloodingArray[2*d*] is the same as the element in FloodingArray[0], then that node will declare itself a cluster head. Lines 18–19 show the second cluster head selection rule to select another node as a cluster head. Like the function pair, a node compares *d* values from FloodingArray[1] to FloodingArray[*d*] with *d* values from FloodingArray[*d* + 1] to FloodingArray[2*d*], to determine whether they are the same value. Line 20 describes the third cluster head selection rule. The node selects another node with the flooding value in Floodmax-*d* as the 0*^th^* round value. The function CH_ID returns the cluster heads’ ID as a result of cluster head selection rules 2 and 3.

### 3.3. Validation of the Proposed Method and the Effect of Weight Factor (ω)

If the flooding value selected in Floodmin-2*d* is the value computed in the 0*^th^* round, the node will declare itself a cluster head during cluster head selection. This node has higher residual energy or degree, since its flooding value is selected in Floodmax-*d*. This is clear, if we examine the process of cluster creation. Nodes selected as cluster heads can be classified into two types. The first class includes nodes with the maximum flooding value within *d*-hop. The second class includes nodes with the maximum flooding value among *d*-hop neighbors’ neighbors.

Consider node *x* in the first class; node *x* updates the flooding values of its *d*-hop neighbors during FloodMax. Therefore, all nodes within the *d*-hop coverage area of node *x* will record the flooding value of node *x* in their FloodingArray. At the conclusion of Floodmin, node *x* will declare itself a cluster head and some *d*-hop neighbors will select node *x* as their cluster head. Consider node *y* in the second class. Although flooding value of node *y* in its FloodingArray is changed by a larger flooding value, its flooding value in the 0*^th^* round continues to propagate and consumes all of the smaller node’s flooding values within its *d*-hop neighbors. Therefore, at the completion of Floodmin, node *y* receives its flooding value in the 0*^th^* round again. Every node in the second class will elect themselves as cluster heads based on the first cluster head rule, and some *d*-hop neighbors will choose node *y* as their cluster head. Hence, any node that survives the Floodmax stage will elect itself as a cluster head. That is, Floodmax enables us to select favorable nodes with a higher flooding value. This allows a stable backbone to be constructed and balances the energy consumption around the cluster heads for WSNs.

**Figure 3.**
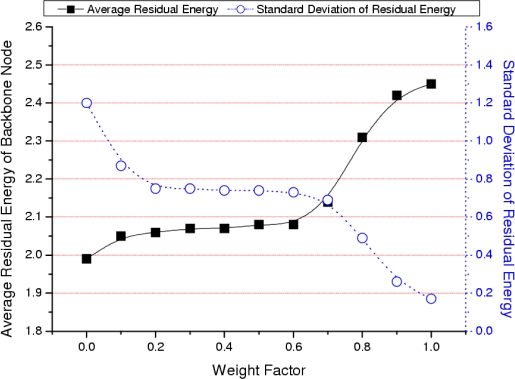
Average and standard deviation of residual energy.

**Figure 4.**
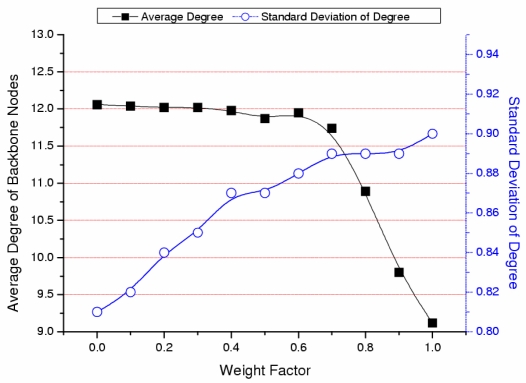
Average and standard deviation of degree.

We simulate our algorithm to confirm that the backbone is composed of nodes with higher energy and degree. The parameters of the simulation are shown in Table 2 of Section 4. Figure 3 shows the results of the average and standard deviation of the backbone nodes’ residual energy as the weight factor changes. As the weight factor approaches 1, the weight of the nodes’ residual energy increases and the average residual energy of the backbone nodes also increases (solid line in Figure 3). As the weight factor approaches 1, nodes with higher residual energy are selected as cluster heads in advance and the distribution of residual energy among backbone nodes is even (dotted line in Figure 3). Figure 4 shows the average and standard deviation of degree of backbone nodes as the weight factor changes. As the weight factor approaches 0, the weight of the node’s degree increases and the average degree of the backbone nodes also increases (solid line in Figure 4). As the weight factor approaches 0, the nodes with higher degree are selected as cluster heads in advance. The distribution among degree of backbone nodes could be even (dotted line in Figure 4).

**Figure 5.**
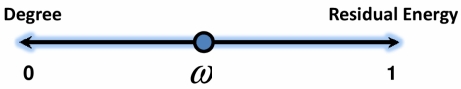
The effect of weight factor (*ω*) on the backbone.

This weight policy can be implemented to optimize in various environments of WSNs by changing the weight factor, *ω*. As shown in Figure 5, we could create an appropriate backbone using the flooding value. For example, in some WSNs, a cluster head frequently handles heavy inter-cluster traffic and coordinates many nodes. In this case, a longer expected lifetime for sensor networks can be achieved by giving a weight factor value approaching 1. Although cluster heads consume more energy than ordinary nodes, the network lifetime could be extended due to their higher residual energy. Alternatively, assuming that each node periodically transmits the sensing information to the cluster head in a multi-hop manner, the nodes near the cluster head tend to exhaust their energy sooner due to the energy consumption in forwarding packets. In this scenario, a value approaching 0 is assigned to the weight factor. Since cluster heads have higher degree, MCBT balances energy consumption by distributing the traffic load among nodes around the cluster head.

## 4. Performance Evaluation

### 4.1. Simulation Environment

Via the ns-2 simulator, we implement and compare the performance of MCBT with other backbone construction schemes, i.e., HCDD and MMM. Their information is listed in Table 1 (in Table 1, *d* is the maximum number of hops between the cluster head and ordinary node and *n* is the number of nodes in the network). The MAC layer follows the IEEE 802.11 MAC specification.

The main objective of our scheme is to create a stable backbone with the node’s residual energy and degree taken into account, and each node transmits packets through this stable backbone. Thus, we evaluate the performance of the backbone construction scheme in terms of the average residual energy or degree of backbone nodes, the standard deviation of the residual energy or degree of backbone nodes, the average residual energy of backbone nodes after event occurrence, and the network lifetime.

- **Average residual energy or degree of backbone nodes:**
  The residual energy or degree of the backbone nodes shows the stability of the backbone. The demands on the backbone nodes are larger than those of ordinary nodes and nodes around the cluster head consume more energy in order to forward packets. However, by selecting a node with higher energy or degree, the high energy consumption of specific nodes should be alleviated.
- **The standard deviation of residual energy or degree of the backbone nodes:**
  Assume that the number of backbone nodes in the network is *m*, the residual energy or degree of each backbone node is *z*, and the average residual energy or degree of backbone nodes is *z̄*, then the standard deviation is represented by $\sigma =\sqrt{\frac{\sum_{i=1}^{m}{\left(z_{i}-\bar{z}\right)}^{2}}{m-1}}$. The standard deviation of the backbone nodes refers to the distribution rate of backbone nodes’ residual energy or degree. A small standard deviation means that the residual energy or degree of each backbone node is similar to the average value and the residual energy or degree of each backbone node is almost uniform. If the average residual energy or degree of backbone nodes is high and their standard deviation is low, then the node with higher residual energy or degree is selected as a backbone node in advance. Moreover, the backbone is composed of nodes with even characteristics. This may decrease the rapid disconnection of the network caused by a load imbalance.
- **The average residual energy of backbone nodes and the network lifetime after events occur:**
  We can evaluate the network lifetime by measuring the operational time until the backbone is disconnected or by counting the maximum number of transmitted messages from source to sink. This indirectly indicates how long a backbone is maintained before its reconstruction. Reconstruction overhead places a heavy load on the network, and it is important to ensure the backbone is stable and maintained for a long time. The time can be affected by variable factors, such as routing paths and data transfer collisions. Thus, in this paper, we compare the network lifetime with other algorithms by evaluating the maximum number of transmitted messages.

The main parameters of our simulation are listed in Table 2. The sensor nodes are randomly selected as the source and a residual energy below 2.5 J is randomly assigned to each node. Our simulation environment uses the following energy model [24]: *E_tx_* = *α*_11_ + *α*_2_*r^n^, E_rx_* = *α*_12_, where *E_tx_* and *E_rx_* denote the energy consumed to transmit and receive a bit over a distance *r*, respectively. *α*_11_ is the energy/bit consumed by the transmitter electronics. *α*_2_ is the energy dissipated in the transmit op-amp and *α*_12_ is the energy/bit consumed by the receiver electronics.

### 4.2. Simulation Results

#### Influence of Number of Nodes

**Figure 6.**
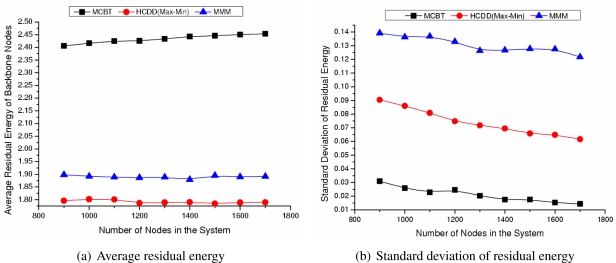
Residual energy of backbone nodes.

**Figure 7.**
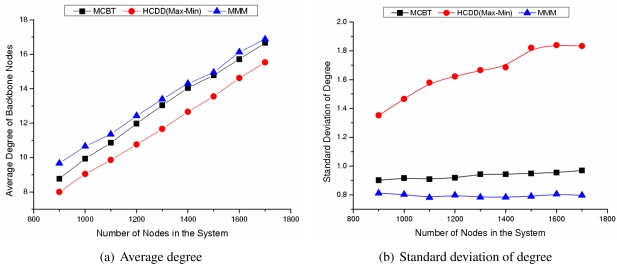
Degree of backbone nodes.

Figure 6 shows the average and standard deviation of the backbone nodes’ residual energy as the number of nodes changes. As shown in Figure 7(a), regardless of the number of nodes composing the network, MCBT (*ω* = 0.7) maintains the highest residual energy. The backbone nodes’ average residual energy of MCBT, MMM, and HCDD are 2.43 J, 1.89 J, and 1.79 J, respectively. MCBT exhibits 22% and 26% improvement respectively, compared with MMM and HCDD. Considering that the backbone nodes have greater demands in terms of packet transmission than those of the ordinary nodes, we can form a stable backbone by selecting nodes with higher residual energy as cluster heads. The standard deviation describes the difference in residual energy among backbone nodes as shown in Figure 7(b). MCBT selects nodes with higher residual energy as cluster heads in advance, so it has a low standard deviation, averaging around 0.01 ∼ 0.03 J.

#### Influence of Number of Events

**Figure 8.**
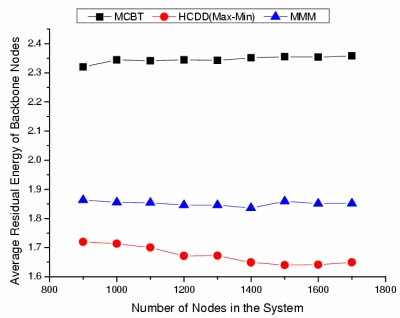
Average residual energy after event occurrence.

Figure 7 shows the average and standard deviation of degree of backbone nodes as the number of nodes changes. In Figure 8(a), when the number of nodes increases, the network density also increases, thus the average degree of the backbone nodes increases. The range among degree of nodes is not high, so the average degree of each algorithm is similar. MMM always performs well, since it forms the backbone by taking into account the node degree. The average degree of backbone nodes of MCBT is similar to that of MMM because MCBT (*ω* = 0.7) considers both degree and energy. If we set *ω* to 0, MCBT has a similar value to MMM. As shown in Figure 8(b), the standard deviations of degree of backbone nodes in MCBT and MMM are very small values, such as 0.93 and 0.79. Nodes around the cluster head consume more energy than nodes located far from the cluster head, since they frequently transmit packets to the cluster head. However, if we select the nodes with high degree as cluster heads, we could distribute the packet forwarding load among nodes around the cluster head, and thus extend the network lifetime.

Figure 8 shows the average residual energy of backbone nodes after 30 sources initiate 20 events periodically. It appears to be a similar graph to that of the average residual energy in the early phase of backbone construction (Figure 7(a)). Therefore, we know that the stability of the backbone of MCBT is maintained after data transmission.

**Figure 9.**
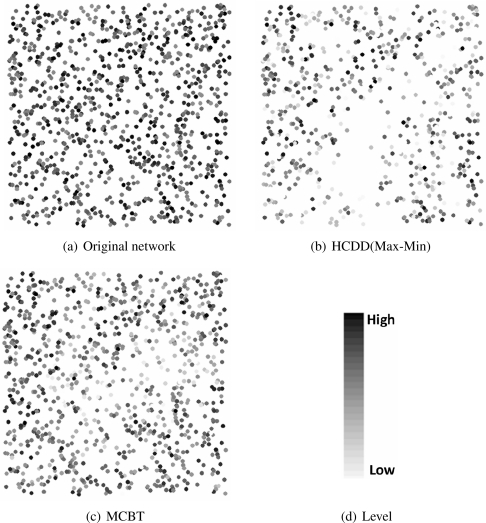
Remaining energy of nodes after event occurrence.

Figure 9 shows the remaining energy of nodes when 30 sources initiate 40 events periodically. The lighter color of the node denotes a lower residual energy. HCDD has large differences in terms of residual energy between, before, and after event occurrence, compared with MCBT. This means the backbone of HCDD is composed of nodes with low energy. Thus, HCDD has many backbone nodes that exhaust energy when many events occur, which causes network disconnection.

**Figure 10.**
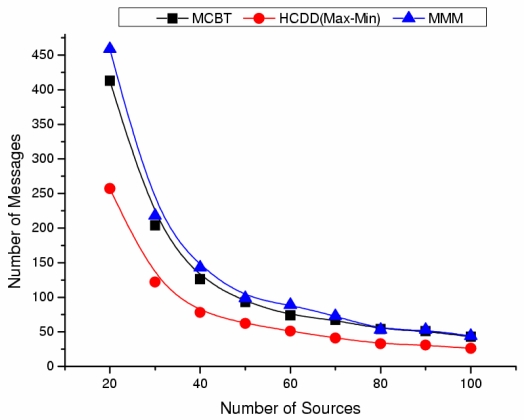
Maximum number of transmission messages.

Figure 10 shows the maximum number of transmitted messages from source to sink before reconstruction of the backbone at different number of sources. The maximum number of transmitted messages is the performance metric to show the network lifetime indirectly. If the path differs for the same source and sink, the time can be affected by network conditions, such as collisions and delays. Therefore, the maximum number of transmitted messages, excluding those factors, is appropriate to measure the network lifetime. As the number of sources increases, the performances of MCBT and other schemes decrease. MMM performs 11% better when the number of sources is small. However, as shown in Figure 10, when the number of sources increases, the difference between MCBT and MMM becomes negligible. The good performance of MMM is due to the selection of cluster heads with a high degree. This provides load distribution among nodes around the cluster head, so the loss of energy due to packet forwarding is decreased. Moreover, the degree based algorithm such as MMM provides high connectivity. Even when the path used at the first time gets disconnected, it will be able to use another path. Therefore, the maximum number of transmitted messages is high. However, by managing the weight factor, MCBT can select nodes with higher degree as the cluster heads, which reflects the advantages of MMM. MCBT appropriately considers both important factors (residual energy and degree) based on system conditions. In addition, MCBT has better time complexity than MMM as shown in Table 1.

## 5. Conclusion

In this paper, we propose a stable backbone tree construction algorithm using multi-hop clusters for WSNs. MCBT constructs the backbone by considering the node’s residual energy and degree. Since the backbone nodes have extra functionality and consume more energy compared with other nodes in the network, the construction of a stable backbone by selecting nodes with higher energy as the cluster heads could prolong the network lifetime. Nodes around the cluster head also consume more energy to forward packets, thus selecting the node with higher degree could distribute the load for packet forwarding among the nodes near the cluster head and balance the energy consumption. Moreover, MCBT could create an appropriate backbone for the system by adjusting the weight factor. Simulations show that MCBT performs better than existing schemes, in terms of the average and the standard deviation of residual energy or degree of backbone nodes, the average residual energy of backbone nodes after event occurrence, and the network lifetime.

## Figures and Tables

**Table 1.** Three clustering schemes in simulation.

Schemes	Cluster head selection criteria	Time complexity
HCDD	ID	O (*d*)
MMM	Degree	O (*n*)
MCBT	Energy&Degree	O (*d*)

**Table 2.** Simulation parameters.

Network size	500 m × 500 m
Transmission range	28 m
Initial energy	2.5 J
Data packet size	500 bytes
Control packet size	15 bytes
Energy consumption model *E_tx_*	*α*_11_ + *α*_2_*r^n^*
Energy consumption model *E_rx_*	*α*_12_
*α*_11_*, α*_12_	80 nJ/bit
*α*_2_	1 pJ/bit/m^2^
*n*	2
